# FANCD2 re-expression is associated with glioma grade and chemical inhibition of the Fanconi Anaemia pathway sensitises gliomas to chemotherapeutic agents

**DOI:** 10.18632/oncotarget.2225

**Published:** 2014-07-16

**Authors:** Abhijit A. Patil, Parag Sayal, Marie-Lise Depondt, Ryan D. Beveridge, Anthony Roylance, Deepti H. Kriplani, Katie N. Myers, Angela Cox, David Jellinek, Malee Fernando, Thomas A. Carroll, Spencer J. Collis

**Affiliations:** ^1^ Sheffield Cancer Research Centre, Academic Unit of Molecular Oncology, Department of Oncology, University of Sheffield Medical School, Beech Hill Road, Sheffield, UK; ^2^ Neuro-Oncology Group, Sheffield Teaching Hospitals NHS Foundation Trust, Royal Hallamshire Hospital, Sheffield, UK; ^3^ Department of Histopathology, Sheffield Teaching Hospitals, Royal Hallamshire Hospital, Sheffield, UK

**Keywords:** Glioma, Glioblastoma, Fanconi Anaemia, Temozolomide, Chemo-sensitisation

## Abstract

Brain tumours kill more children and adults under 40 than any other cancer. Around half of primary brain tumours are glioblastoma multiforme (GBMs) where treatment remains a significant challenge. GBM survival rates have improved little over the last 40 years, thus highlighting an unmet need for the identification/development of novel therapeutic targets and agents to improve GBM treatment. Using archived and fresh glioma tissue, we show that in contrast to normal brain or benign schwannomas GBMs exhibit re-expression of FANCD2, a key protein of the Fanconi Anaemia (FA) DNA repair pathway, and possess an active FA pathway. Importantly, FANCD2 expression levels are strongly associated with tumour grade, revealing a potential exploitable therapeutic window to allow inhibition of the FA pathway in tumour cells, whilst sparing normal brain tissue. Using several small molecule inhibitors of the FA pathway in combination with isogenic FA-proficient/deficient glioma cell lines as well as primary GBM cultures, we demonstrate that inhibition of the FA pathway sensitises gliomas to the chemotherapeutic agents Temozolomide and Carmustine. Our findings therefore provide a strong rationale for the development of novel and potent inhibitors of the FA pathway to improve the treatment of GBMs, which may ultimately impact on patient outcome.

## INTRODUCTION

Brain tumours are the biggest cancer killers of the under 40s with over 400,000 new cases diagnosed worldwide each year. They also represent the commonest site for solid tumours in childhood under the age of 15 [1]. However, brain tumour research has received far less funding than many other cancers and patient survival has improved little over the last 40 years (Cancer Research UK, Brain Tumour Research and The Brain Tumour Charity). There is therefore an unmet need for the development of novel targets and agents to improve GBM treatment.

Around half of primary brain tumours are high-grade malignant astrocytomas, also known as glioblastoma multiforme (GBM). Treatment of GBM remains a significant challenge with a median patient survival of ~1yr. Current treatments are limited by anatomical constraints e.g. proximal neural areas, high resistance to radio-/chemotherapy and the blockading effect of the blood-brain barrier to systemically administered agents [1]. In addition to surgery, standard treatment for these tumours consists of chemotherapeutic agents such as Temozolomide (TMZ), Carmustine (BCNU) and/or radiotherapy regimes. Previous work has suggested that up-regulation of DNA response (DDR) pathways in GBM may contribute to some of the relative ineffectiveness of these agents [2, 3]. However, the underlying molecular mechanisms driving inherent or acquired resistance of GBM to DNA damaging agents has yet to be determined. There is therefore precedence for determination of expression levels/activity of DDR factors in GBM that could account for resistance to therapeutic agents, as well as highlighting novel drug targets as a means to augment the cytotoxicity of current therapeutic regimes.

The Fanconi Anaemia (FA) pathway is comprised of at least 16 proteins that promote the repair of lesions that impede DNA replication in order to preserve genome integrity [4-6]. As such, the FA pathway is activated during DNA replication or in response to the detection of replication-blocking lesions [7], and is therefore often down regulated in differentiated cells [8]. Consistent with this, previous studies have shown that highly proliferative tissues such as testis, tonsils and spleen exhibit high levels of FANCD2 and FANCA expression, yet non-proliferative tissues such as lung, liver, muscle and the brain exhibit minimal protein expression [9, 10]. Furthermore, a recent large-scale gene expression study revealed that at least 11 of the FA genes exhibit increased expression at the mRNA level in GBM compared to normal brain tissue [11]. This raises the possibility that as brain tumours such as oligodenrogliomas and astrocytomas become more proliferative, they re-activate the FA pathway.

The FA pathway is activated in response to a variety of DNA damaging agents that impede DNA replication, where it acts to facilitate lesion repair and/or bypass, and co-ordinates recruitment and activity of other DNA repair pathways needed to repair such lesions [12]. As such, cells defective in the FA pathway are rendered sensitive to a variety of DNA crosslinking agents, making the FA pathway an attractive target for inhibition in cancer cells. In an attempt to identify novel inhibitors of the FA pathway (FAPi), several groups have carried out large-scale small-molecule library screens [13-16]. These studies have revealed four FAPi that are currently commercially available; curcumin and its monoketone analogues EF-24 & 4H-TTD, and the menadione analogue DDN [13-16]. However, the mode of action of these compounds has not been elucidated to date, and curcumin may impact on multiple DNA repair pathways [17]. Pertinent to the treatment of GBM, the FA pathway is activated in response to alkylating agents such as Temozolomide (TMZ) and Carmustine (BCNU; [18]), and previous work has demonstrated that FA-deficient cells are more sensitive to TMZ and BCNU than FA-proficient cells [18, 19]. Given these previous findings, we hypothesised that the FA pathway would not be expressed/active in normal non-proliferative brain tissue, but be re-activated in rapidly proliferating GBMs. As such, FA pathway inhibition in aggressive brain tumours may offer an exploitable therapeutic window to improve the treatment of GBM to currently used chemotherapeutic agents.

Using both archived and fresh GBM tissue, we demonstrate that in contrast to normal brain tissue or benign Schwannomas, the key FA pathway protein FANCD2 is re-expressed and that the FA pathway is active in GBMs. Furthermore, expression levels of FANCD2 strongly correlate with tumour grade. We also show that three separate small molecule FA pathway inhibitors sensitise GBMs (primary and established cultures) to both TMZ and BCNU irrespective of MGMT status. Our data therefore highlights a potential exploitable therapeutic window to increase the cytotoxicity of chemotherapeutic agents used to treat GBM, whilst sparing normal non-cancerous brain tissue.

## RESULTS

### The FA pathway is re-expressed and active in high-grade GBM, with FANCD2 protein expression associated with tumour grade

**Figure 1:**
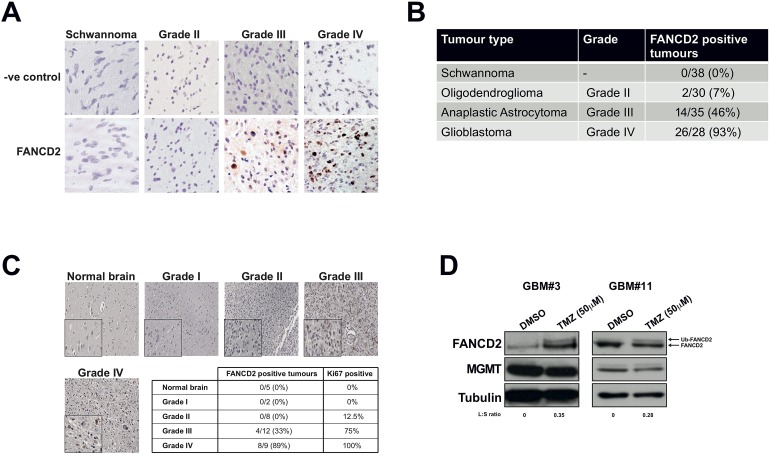
The FA pathway is highly expressed and active in high-grade gliomas, with FANCD2 expression associated with tumour grade A: Representative images of FANCD2 staining in archived FFPE sections of schwannoma (negative control) and gliomas of various tumour grades as indicated. FANCD2 is a nuclear protein, which can be seen in the grade III and grade IV tumours shown (brown DAB staining). B: Quantification of FANCD2 expression in 131 schwannoma and gliomas of various tumour grades. A chi-squared test was used to test for association with tumour grade. C: Representative images of FANCD2 staining in normal brain and gliomas of various tumour grade taken from commercial tissue microarray. Insert at bottom left of each image shows a magnified field from the section to show nuclear FANCD2 staining. The table underneath shows quantification of FANCD2 staining in the 36 samples present on the tissue microarray, indicating number of each sample exhibiting FANCD2 staining and the percentage of each tumour grade positive for FANCD2 expression. The percentage of FANCD2 positive tumours that also stained positive for Ki67 (supplementary Figure S1B) is also shown in the table. A chi-squared test was used to test for association with tumour grade. D: Western blots showing FANCD2 expression and activation (mono-ubiquitinated FANCD2; Ub-FANCD2) following Temozolomide (TMZ) treatment in two independently derived primary glioma cultures. To determine FA pathway activation, the normalised ratio of Ub-FANCD2 (L) to FANCD2 (S) was calculated for TMZ vs DMSO treated cells as previously described [30], and is shown under the blots as an L:S ratio. Also shown is MGMT expression and tubulin, which serves as a loading control.

Given that the FA pathway responds to lesions that impede on-going DNA replication [12], is down-regulated in differentiated cells [8], expressed in highly proliferative tissue, but not expressed in normal brain tissue [9, 10]; we hypothesised that FA protein expression would be elevated in GBM and might potentially correlate with clinical grade. To test this, we quantified the expression of the key FA pathway protein FANCD2 [6] in 131 archived FFPE glioblastoma specimens collected at the Royal Hallamshire Hospital's neuro-oncology department. As normal brain material is difficult to obtain, benign schwannomas served as a negative control for these experiments (Figure 1A). FANCD2 expressing cells were never detected in schwannomas and only 7% of grade II oligodendrogliomas exhibited FANCD2 expression (Figures 1A and 1B). However, 46% and 93% of grade III and grade IV gliomas respectively stained positive for FANCD2 expression (Figures 1A and 1B). Comparable data was obtained using a commercial tissue microarray, in which normal brain tissue was devoid of any FANCD2 expression, and expression was more prevalent with increasing tumour grade (Figure 1C). In both experiments there was a significant association between the presence of FANCD2 expression and tumour grade (p<2×10^−16^ and p<1×10^−4^ respectively). Given these findings, we attempted to stratify overall survival of the Grade III patients with FANCD2 staining, but did not detect a significant correlation (data not shown). However, this cohort was very heterogeneous with some patients having undergone aggressive de-bulking surgery and radiotherapy, while others having just biopsy. Therefore, given the relatively small number of cases in this cohort and the differences in treatment regimes among them, it is perhaps not surprising that no prognostic correlation between FANCD2 staining and patient survival could be ascertained at this time.

Consistent with the overall findings in our archived tissue samples, we observed negligible FANCD2 staining in one particular low-grade (radiology diagnosis) oligodendroglioma originally diagnosed in 2011 (supplementary Figure 1B). This tumour was graded WHO II at biopsy later that year and immunohistochemical analyses revealed weak FANCD2 expression (supplementary Figure 1B). This patient was re-admitted in 2012 with biopsy proven transformation to grade III anaplastic astrocytoma from which we were again able to determine FANCD2 expression. Interestingly, the patient's grade III tumour exhibited increased FANCD2 expression compared to the previous biopsy proven lower grade disease (supplementary Figure 1B). Even with the caveat that this is a single case, taken together with our analyses of over 165 normal, benign schwannomas and brain tumours derived from multiple patients (Figures 1A-C), our data reveal that FANCD2 expression is significantly associated with tumour grade. These findings therefore raise the possibility that the FA pathway is more active in high-grade gliomas, and may therefore contribute to chemotherapeutic resistance.

Even though we have determined that FANCD2 expression increases with brain tumour grade, it does not indicate if the FA pathway is active in these tumours and could contribute to chemotherapeutic resistance mechanisms [19, 20]. To determine if the FA pathway is indeed active in high-grade glioma, we derived primary cultures from freshly resected grade III/IV gliomas (supplementary Figure 1C), using previously described protocols [21, 22]. In agreement with our findings in archived FFPE gliomas, all successfully established primary GBM cell cultures to date were found to express FANCD2 (Figure 1D and data not shown). Importantly, the FA pathway is active in these cells, as treatment of these primary cultures with the chemotherapeutic agent Temozolomide (TMZ) activated mono-ubiquitination of FANCD2 and re-localisation to nuclear foci (Figures 1D and supplementary Figure S1D). These are two key steps in activation of the FA pathway [23], and imply that the rest of the FA pathway is also expressed to sufficiently high levels to facilitate FANCD2 mono-ubiquitination/re-localisation. Overall, these data are consistent with previous mRNA studies [11], and demonstrate that FANCD2 protein expression increases with glioma grade and is active and responsive to chemotherapeutic agents. Given that normal brain tissue [9, 10], or benign tumours derived from a non-glial lineage (Figures 1A-B) do not express FANCD2, we postulate that inhibition of the FA pathway in high-grade gliomas represents a novel therapeutic window to improve the treatment of these incurable tumours.

### Small molecule FA pathway inhibitors disrupt FA pathway activation in both established and primary glioblastoma cell cultures

Previous studies have demonstrated that the FA pathway is activated in response to chemotherapeutic agents currently used to treat GBM such as the alkylating agents Temozolomide (TMZ) and BCNU [18], and others have shown that FA-deficient cells are more sensitive to these agents than FA-proficient cells [18, 19]. However, the effects of several commercially available FA inhibitors (FAPi) in GBM cells have not been determined. We therefore used the glioma cell lines U87 and U138, which are respectively proficient and deficient for the FA pathway [18], to ascertain if FAPi can sensitise GBM cells to the chemotherapeutic agents currently used to treat high-grade gliomas. To date, four separate FAPi have been described that are commercially available; curcumin, its monoketone analogues EF-24 & 4H-TTD, and the menadione analogue DDN [13-16]. However, in initial toxicity studies, we found that 4H-TTD was toxic to both U87 and U138 cells even at nM concentrations (data not shown), and was therefore excluded from our further analyses, as was the recently reported FAPi Ouabain [24].

**Figure 2:**
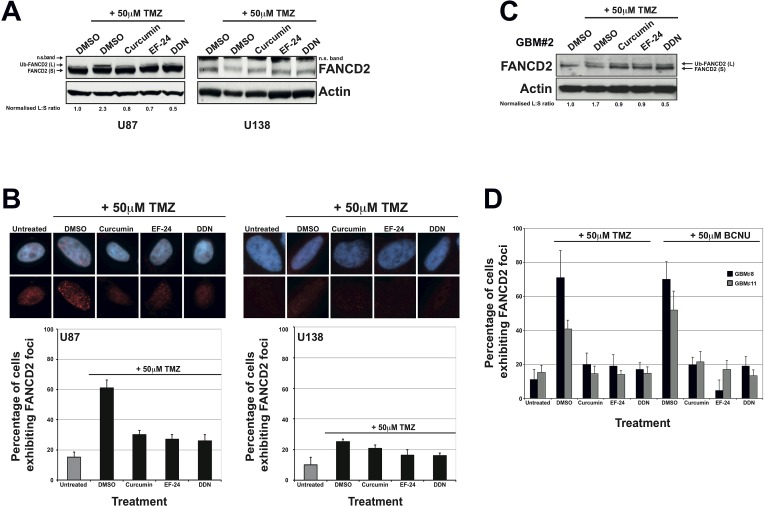
The FAPi curcumin, EF-24 and DDN inhibit FA pathway activation in immortalised and primary glioma cell cultures **A**: Representative western blots showing FANCD2 and actin (loading control) expression in U87 (left panel) and U138 cells (right panel). Treatment with Temozolomide (TMZ) activates the FA pathway as evidenced by the appearance of a slower migrating form of FANCD2 with represents the mono-ubiquitinated form (Ub-FANCD2). Pre-treatment with the indicated that FAPi severely inhibits FA pathway activation as evidenced by significantly reduced Ub-FANCD2 after TMZ treatment. DMSO treatments were used as negative controls. To determine FA pathway activation, the normalised ratio of Ub-FANCD2 (L) to FANCD2 (S) (normalised to DMSO+TMZ treated cells) was calculated for each treatment as previously described [30] and shown under the blots for U87 cells. Note that all values are zero for U138 cells). As described previously, U138 cells express low levels of FANCD2 and exhibit a defective FA pathway (no Ub-FANCD2 after TMZ treatment). The FANCD2 antibody recognises a non-specific band in U138 cell line as indicated (n.s. band), which is most likely due to reduced epitope expression in this cell line. **B**: Immunofluorescence detection and quantification of cells exhibiting 10 or more nuclear FANCD2 foci (a marker of an active FA pathway) in U87 and U138 cells treated as described above. Upper panel shows representative images for treated cells and the graph below show quantified data from at least three independent experiments. Error bars represent standard deviation of the means. **C**: Western blot showing FANCD2 expression and activation in a primary glioma culture. As with immortalised glioma cell lines, pre-treatment of the primary culture with the indicated FAPi compromises FA pathway activation as calculated by determining the normalised ratio of Ub-FANCD2 (L) to FANCD2 (S) as described in panel A. **D**: Quantification of FANCD2 nuclear foci formation in 2 separate primary glioma cultures. Cells were either treated with DMSO (negative control) or TMZ, with or without pre-treatment with the indicated FAPi as described in the material and methods. Representative images for this data are shown in supplementary Figure S1D. Due to the difficulty in the long-term propagation of such cultures and their weak adherence on glass coverslips, error bars represent standard deviation of the means from 2 replica plates.

Treatment of U87 cells with non-toxic doses of curcumin, EF-24 or DDN resulted in significant inhibition of FANCD2 mono-ubiquitination following treatment with TMZ (Figure 2A). This was accompanied by a concurrent reduction in the number of cells exhibiting FANCD2 nuclear foci (Figure 2B). Consistent with a previously reported defective FA pathway [18], the glioma cell line U138 displayed reduced endogenous levels of FANCD2 and an inability to activate the FA pathway following TMZ treatments (Figures 2A and 2B). Comparable data was obtained in both U87 and U138 cells using BCNU in place of TMZ (supplementary Figure S2A). It is important to note that the inhibitory effects on FA pathway activation conferred by curcumin, EF-24 or DDN were not a consequence of altered cell cycle distribution (data not shown). As well as serving as an important negative control for these experiments, the data generated in U138 cells suggest that perturbation to FA pathway activity in U87 cells was a direct consequence of FAPi treatments, which was further confirmed using isogenic cell lines (see later).

To ascertain if FAPi could inhibit the activation of the FA pathway observed in primary glioma cultures (Figure 1D), we treated several independently derived primary GBM cell cultures with TMZ with or without the prior treatment of FAPi. Consistent with results obtained in U87 cells, treatment of primary glioma cell cultures with curcumin, EF-24 and DDN all resulted in significant inhibition of FA pathway activation in response to Temozolomide treatments as measured by both reduced mono-ubiquitination of FANCD2 (Figure 2C) and a reduced ability to form FANCD2 nuclear foci (Figures 2D and S1D). Together with the data presented in Figure 1, these results provide strong evidence that unlike normal brain tissue, an active FA pathway is highly expressed in high-grade gliomas and the use of FAPi may therefore sensitise these inherently resistant tumours to chemotherapeutic agents.

### Inhibition of the FA pathway sensitises glioblastoma cells to chemotherapeutic agents irrespective of MGMT status

**Figure 3:**
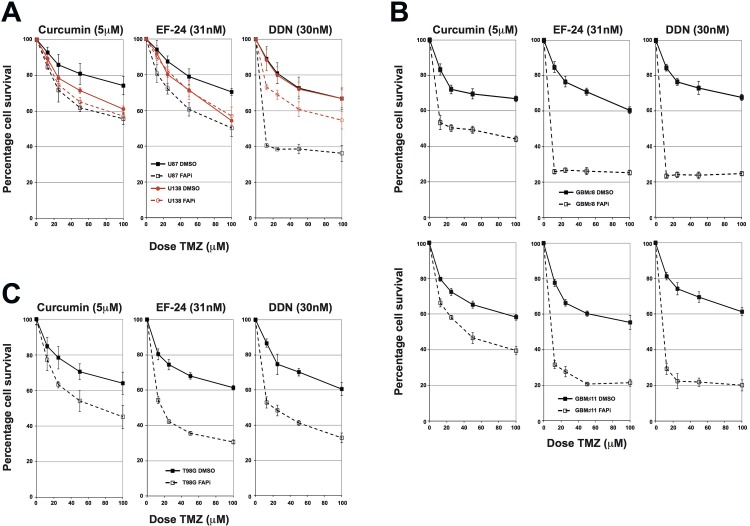
FAPi sensitise immortalised and primary glioma cultures to chemotherapeutic agents irrespective of MGMT status **A**: MTT Temozolomide cytotoxicity assays for U87 and U138 cells treated with the FAPi curcumin, EF-24 or DDN. Solid lines represent cells pre-treated with DMSO and dashed lines represent cells pre-treated with the indicated FAPi. Data shown is the mean from at least 3 independent experiments with error bars representing the standard deviation of the means. **B**: MTT Temozolomide cytotoxicity assays for two independently derived primary glioma cultures from at least three independent experiments. An additional example is shown in supplementary Figure S2B. Solid, dashed lines and error bars are as described in panel A. C: MTT Temozolomide cytotoxicity assays for T98G cells pre-treated with either DMSO or the FAPi curcumin, EF-24 or DDN. Solid, dashed lines and error bars are as described in panel A.

To determine if inhibition of the FA pathway could sensitise glioma cells to chemotherapeutic agents, we carried out cytotoxicity assays, initially using U87 (FA proficient) and U138 (FA deficient) cells. Cells were pre-treated with the FAPi curcumin, EF-24 or DDN, and then treated with increasing doses of either TMZ or BCNU. All three FAPi conferred a significant increased sensitivity to both TMZ and BCNU in the FA-proficient U87 cells (Figure 3A and supplementary Figure S2B respectively). Importantly, this increased sensitivity could be mainly ascribed to inhibition of the FA pathway, rather than any non-specific activities of the FAPi, as comparable increased sensitivity was not observed in FA-deficient U138 cells treated with either curcumin of EF-24, with only a modest increase observed in DDN treated cells (Figure 3A and supplementary Figure S2B). In order to ascertain if inhibition of the FA pathway would also confer a chemo-sensitising phenotype to primary glioma cultures, we repeated these experiments using several primary glioma cultures derived from fresh tissue (supplementary Figure 1C). Consistent with data obtained in immortalised glioma cell lines (Figures 3A and S2B), and with the fact that FAPi are capable of inhibiting FA pathway activation in primary glioma cell lines (Figures 2C, 2D and S1D); pre-treatment with curcumin, EF-24 or DDN all led to significantly increased TMZ sensitivity in several independently derived primary GBM cultures (Figure 3B and supplementary Figure S2C). As with immortalised glioma cell lines, both EF-24 and DDN were generally greater chemo-sensitisers than curcumin, which likely reflects their increased potency to inhibit the FA pathway [15, 16].

Inherent resistance of high-grade gliomas to alkylating agents has often been linked to expression of the de-alkylating enzyme MGMT, which is not expressed in around half of these tumours [1]. However, conclusive evidence that such genetic differences account for the majority of tumour cell responses to such agents is still lacking and may also be linked to other DNA repair mechanisms [2]. If inhibition of the FA pathway is to offer promise as a potential chemo-sensitising strategy, then it is important to determine if they confer this function irrespective of MGMT status. U87 cells are MGMT negative and U138 cells are MGMT positive (supplementary Figure S2D), therefore FAPi are capable of sensitising MGMT negative cells to both TMZ and BCNU (Figures 3A and S2B). To determine if they also act as potent chemo-sensitisers in an MGMT positive genetic background, we carried out cytotoxicity assays with the FAPi curcumin in T98G cells, which, like U87 cells are chemo-resistant, yet are MGMT positive (supplementary Figure S2D). Pre-incubation of T98G cells with curcumin, EF-24 or DDN all sensitised T98G cells to TMZ in a similar manner to that observed in the MGMT negative cell line U87 (Figure 3C). We therefore conclude that inhibition of the Fanconi Anaemia pathway offers promise as a chemo-sensitising strategy irrespective of MGMT status, which is an important finding for the future clinical development of FAPi.

**Figure 4:**
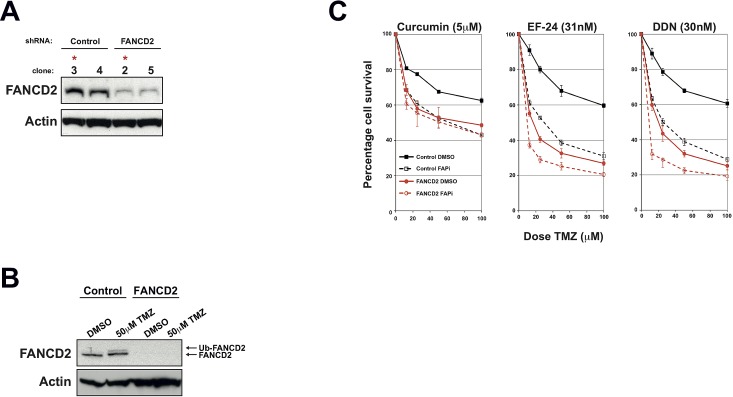
Generation of isogenic FANCD2 proficient and deficient U87 cell lines and their use to determine FAPi specificity **A**: Western blots showing FANCD2 expression in a number of stable shRNA expressing clones. Control cells represent those stably transfected with a non-targeting shRNA plasmid, while FANCD2 are clones derived from cells transfected with FANCD2-targeting shRNA plasmids. Actin is shown as a loading control and red asterisks highlight clones selected for use in further studies based on their residual FANCD2 levels. **B**: Western blot showing FANCD2 expression and activation (Ub-FANCD2) following Temozolomide treatment in the selected stable U87 clones outlined in panel A. **C**: MTT Temozolomide cytotoxicity assays for the selected stable U87 clones outlined in panel A pre-treated with either DMSO or the FAPi curcumin, EF-24 or DDN. Data shown was derived from at least three independent experiments and solid, dashed lines and error bars are as described in Figure 3A.

The use of FA-proficient and FA-deficient U87 and U138 GBM cell lines provides strong proof of concept that the observed increased sensitivity to chemotherapeutic agents is specifically due to inhibition of the FA pathway. However, other genetic differences between these two cell lines could account for some of the phenotypic differences observed, as could potential off-target effects of these FAPi [17, 25]. To provide more conclusive proof and to act as important tool for the further development of FAPi, we generated isogenic U87 cell lines that are proficient or deficient in the FA pathway. To achieve this, we stably integrated plasmids expressing previously validated FANCD2-directed shRNA and screened several clones derived from two independent shRNA or non-targeted control shRNA for FANCD2 levels (data not shown). A cell line was selected that exhibited minimal residual FANCD2 expression and severely compromised FA pathway activation when compared to cell lines stably expressing non-targeted control shRNA (Figure 4A and 4B respectively). As in parental U87 cells, pre-treatment with the FAPi curcumin, EF-24 and DDN sensitised the stable cell line expressing a non-targeting control shRNA to TMZ treatments (Figure 4C). Importantly however, these FAPi had minimal effect of TMZ sensitisation in the cell line depleted of FANCD2 (Figure 4C). Similar data was obtained using an additional stable FANCD2-depleted cell line expressing shRNA targeting a different region of the FANCD2 mRNA (supplementary Figure S2E). The slight increased TMZ sensitivity observed in the FANCD2-depleted cells following pre-treated with either EF-24 or DDN likely reflects their more potent activity towards the residual levels of FANCD2 compared to Curcumin [15, 16]. Collectively, these findings provide strong proof of concept data that FAPi could sensitise gliomas to chemotherapeutic agents by exploiting their heightened FA pathway expression/activity compared to the surrounding normal brain tissue.

## DISCUSSION

We report here the first appraisal of the expression and activity of the FA pathway in high-grade glioma, demonstrating a strong association between FA protein expression and clinical grade. Given that the FA pathway is activated during normal S-phase transit (unperturbed DNA replication) or in response to replication-blocking lesions [4, 5, 7], increased FANCD2 expression in high-grade glioma is most likely a consequence of an increased proliferative index, which also increases with glioma grade. Indeed, analyses of the proliferation marker Ki67 in GBM tissue micro-arrays agree with this hypothesis (Figures 1C and S1C). It is interesting to note that in contrast to these findings, a previous study showed that a cohort of breast tumours exhibiting reduced cytoplasmic FANCD2 expression were correlated with poor survival outcomes [26]. However, our findings are also consistent with previous reports showing that highly proliferative tissues such as testis, tonsils and spleen exhibit high expression of FANCD2 and FANCA, yet non-proliferative tissues such as lung, liver, muscle and the brain exhibit minimal expression of these proteins [9, 10]. We also reveal that FANCD2 expression is negligible in normal brain tissue, which is consistent with previous findings that the FA pathway is down regulated in differentiated cells [8]. Also relevant to our findings is a recent large-scale gene expression study which revealed that at least 12 of the currently 16 known FA genes exhibit increased expression at the mRNA level in GBM compared to normal brain tissue [11]. Taken together, the data presented here are in agreement with previous studies and reveal an exploitable therapeutic window for FA pathway inhibition in high-grade gliomas. In this regard, and as part of our on-going studies towards such clinical application of FA pathway inhibitors, we also report here the first use of multiple commercially available FAPi (curcumin, EF-24, DDN) to sensitise glioma cells to chemotherapeutic agents irrespective of genetic background.

An important aspect to bear in mind when considering clinical applications of FA pathway inhibitors is how such FAPi will be delivered to prevent/minimise possible systemic side effects. The recent interest and advancements in nano-particles combined with magnetic-mediated delivery/localisation is a potentially attractive future approach for drug delivery in glioma. Indeed, it has been recently reported that curcumin and Temozolomide could be successfully co-loaded onto such particles and delivered to both 2D and 3D model cell cultures [27]. Alternatively, oral delivery of an FAPi such as curcumin may offer a viable and safe delivery mechanism [28], assuming of course that sufficiently amounts of active compound at the tumour site could be achieved to confer a chemo-sensitising effect.

Perhaps the most promising approach would either be the use of current intrathecal pumps to facilitate exploitation of the blood-brain barrier to deliver FAPi directly into the cerebrospinal fluid or alternatively intratumoural/intracerebral convection enhanced delivery [29]. Thus, delivery of small-molecule FA pathway inhibitors directly to the cerebrospinal fluid or directly into the brain parenchyma may maximise their sensitising properties at tumour sites, whilst taking advantage of the blood-brain barrier to retain the FAPi agent to minimise possible systemic side effects. Such an approach would therefore facilitate both tumour localisation and reduce potential undesired side effects.

Given that the mechanisms of action for any of the commercially available FAPi described here (curcumin, EF-24 and DDN) are currently unknown; as part of the future development of more potent and specific FAPi, we are presently working with medicinal chemists to develop novel small molecule inhibitors of the FA pathways for which we know the mechanism of action. The isogenic FANCD2 proficient/deficient U87 cells lines described here, along with others that we are presently generating will be instrumental in determining FAPi specificity and for target validation studies. To facilitate this potential future application of FAPi, we are also currently carrying out *in vivo* studies based on the promising *in vitro* findings presented here, using both commercially available FAPi and our novel FAPi in combination with chemotherapeutic agents currently used in the clinical management of high-grade gliomas. Given the poor prognosis of patients with high-grade gliomas and the current unmet need for new therapies for this devastating disease, it is hoped that the data presented here and future *in vivo* studies will facilitate the development of an early phase clinical trial to allow the assessment of the use of FAPi to improve our current treatment of these tumours.

## MATERIALS AND METHODS

### Primary and archived brain tumour samples

Primary brain tumour tissue surplus to clinical requirements was collected from patients attending the Neuro-oncology unit at the Sheffield Royal Hallamshire Hospital, and FFPE tissue was retrieved from the Royal Hallamshire Hospital diagnostic archive. The research involving patient material was approved by the Leeds East Research Ethics Committee (REC reference 11/YH/0319). The tissue microarray containing both healthy and cancerous human brain tissue cores was purchased from Insight Biotechnology. All archived brain tissue were verified for tumour grade by a consultant pathologist (MF).

### Cell Culture

Genetically authenticated U87, U138 and T98G cells were obtained from ATCC and maintained as adherent monolayer cultures in DMEM media containing 10% FBS at 37°C in a humidified atmosphere of 5% carbon dioxide, and sub-cultured when ~70% confluence was reached. Both serum-free and serum-adapted cell cultures derived from primary GBM tissue were prepared as previously described [21, 22] under appropriate ethics approval (REC reference 11/YH/0319). Stable FANCD2-deficient U87 cells were created using the HuSH shRNA system from Origene as described in the manufacturers protocol.

### Antibodies

FANCA (IHC: ab5063, 1:100), FANCD2 (IHC: ab108928, 1:200; IF: ab2187, 1:1000; WB: ab12450, 1:5000) and MGMT (ab39253, 1:1000), Ki67 (ab16667, 1:100). For Western blotting, primary antibodies were visualised using HRP-conjugated anti-rabbit and anti-mouse secondary antibodies at 1:5000 (DAKO P0399 and P0447 respectively). For immunofluorescence, anti-mouse Alexa-488 or anti-rabbit Alexa-594 (Invitrogen) were used at 1:1000. The FANCD2 antibody for IHC was optimised using mouse spleen and brain as positive and negative controls for protein expression respectively (supplementary Figure S1A) as previously described [9], together with FANCD2 proficient and siRNA-depleted cells (data not shown).

### Drug treatments

Cells were treated with Curcumin (5μM), EF-24 (31nM), DDN (30nM) 8 hours prior to treatment with 50μM of either TMZ or BCNU for 24hrs (western blots and immunofluorescence detection of FA pathway activation) or 5 days (cytotoxicity assays).

### Cell lysis and Western Blotting

For whole-cell extracts, cells were solubilized on ice for 20 minutes in lysis buffer; 20 mM Tris-HCl pH 7.5, 150 mM NaCl, 1% Triton X-100, 1 mM DTT and 1 mM EDTA supplemented with 50 U/μl benzonase (Novagen), protease and phosphatase inhibitors (Sigma). Cleared lysates were produced by centrifugation of the resulting samples at 16,000 × g for 15 min at 4°C. Gel electrophoresis was performed using the NuPAGE system (Invitrogen). Briefly, samples were resolved on 4-12% Bis-Tris gels in MOPS buffer, transferred to a PVDF membrane which was then probed for the protein of interest using antibodies diluted in PBS containing 5% Marvel and 0.1% Tween-20 (Sigma).

### Immunohistochemistry and scoring

Formalin fixed paraffin embedded (FFPE) samples were deparaffinised in 2 xylene baths for 5-10 minutes each. Slides were then hydrated through descending grades of ethanol (100% to 70%) for 3 minutes each. After washing the slides in running water for 5 minutes, the sections were then immersed in sodium citrate at pH 6 (Sigma) and microwaved for 3 minutes at high temperature and 7 minutes at low temperature for antigen retrieval. The slides were allowed to cool down in solution and then washed in distilled water for 15 minutes. Sections were then soaked in 3% hydrogen peroxide in methanol at 37°C for 30 minutes to block endogenous peroxidase activity. Following 3 washes in distilled water, slides were then incubated for 60 minutes in 10% normal goat serum (Vector Laboratories) and 10% casein in PBS to block non-specific binding sites and then probed with the relevant primary antibodies prepared in PBS containing 2% sera and 2% casein overnight at 4°C. Next day, slides were rinsed in PBS and then incubated for 60 minutes at room temperature in biotinylated goat anti-rabbit secondary antibody (Vector Laboratories) prepared at 1:200 in PBS containing 2% sera and 2% casein. The antigen-antibody complexes were detected using the Vectastain Elite ABC kit (Vector Laboratories) according to manufacturer's protocol, followed by staining with 3,3′-diaminobenzidin (DAB) peroxidase substrate kit, (Vector Laboratories). Sections were then counterstained in Gill's haematoxylin and dehydrated in ascending grades of ethanol before clearing in xylene and mounting under a coverslip using DPX mountant (Fisher). All sections were verified for tumour grade by a consultant pathologist (MF), who also marked the tumour area for scoring. Scoring of FANCD2 staining was carried out blindly by two independent people (AAP and AR), with positive staining indicating that ≥50% cells in the marked tumour area had positively stained nuclei. A chi-squared test was used to test for association with tumour grade.

### Immunofluorescence

Cells were grown on glass coverslips and treated as indicated, then fixed with 3% buffered paraformaldehyde for 10 min at RT, and permeabilized in PBS containing 0.5% Triton X-100 for 5 min at RT. Cells were then incubated with primary antibody for 2 hrs at RT, and detected with a secondary Alexa-488 or Alexa-594 conjugated goat anti-rabbit or anti-mouse IgG. Antibody dilutions and washes after incubations were performed in PBS. DNA was stained with DAPI (1 μg/ml) and coverslips were mounted in Shandon Immu-Mount medium (Thermo). Fluorescence microscopy was performed on a Nikon Eclipse T200 inverted microscope (Melville), equipped with a Hamamatsu Orca ER camera and a 200 W metal arc lamp (Prior Scientific, United Kingdom), with a 60X and 100X objective lens. Images were captured and analyzed using Volocity software (Improvision).

### Flow Cytometric Analyses

Cells were collected using trypsin, pelleted, washed with PBS, fixed in 70% ice-cold ethanol, and stored at −20°C for up to 2 weeks. After thoroughly washing with PBS, to remove any residual ethanol, cells were stained with a propidium iodide solution (50 μg/ml) containing RNase A (25 μg/ml) for 30 min before flow cytometry was performed on a Becton Dickinson FACScalibur instrument. The percentage of cells in each cell cycle phase was subsequently calculated using FloJo analysis software.

### Cytotoxicity assays

Cells were plated at a density of between 1000-2000 cells/well in 96-well plates (depending on cell line), and the following day treated with appropriate non-toxic concentrations of FAPi for 8 hours, after which time, cells were then treated with DMSO, TMZ or BCNU. After 5 days of growth, MTT reagent was added to the cells at a final concentration of 3 mg/ml, and incubated at 37°C for 3 hrs. The media was removed and replaced with 200 μl DMSO to solubilise the formazan product, which was quantified by determining optical density at 540 nm using a spectrophotometric microtitre plate reader. Cytotoxicity was calculated for each treatment by normalisation to appropriate vehicle only (DMSO-treated) controls.

## SUPPLEMENTARY MATERIAL AND FIGURES


