# Supplementary material for: FANCD2 re-expression is associated with glioma grade and chemical inhibition of the Fanconi Anaemia pathway sensitises gliomas to chemotherapeutic agents
Source: Oncotarget. 2014 Jul 16;5(15):6414–24. doi: 10.18632/oncotarget.2225 (PMC4171640; doi:10.18632/oncotarget.2225)
Supplement: Supplementary file 1 [file oncotarget-05-6414-s001.pdf]

FANCD2 re-expression is associated with glioma grade and chemical inhibition of the Fanconi Anaemia pathway sensitises gliomas to chemotherapeutic agents

Supplementary Material

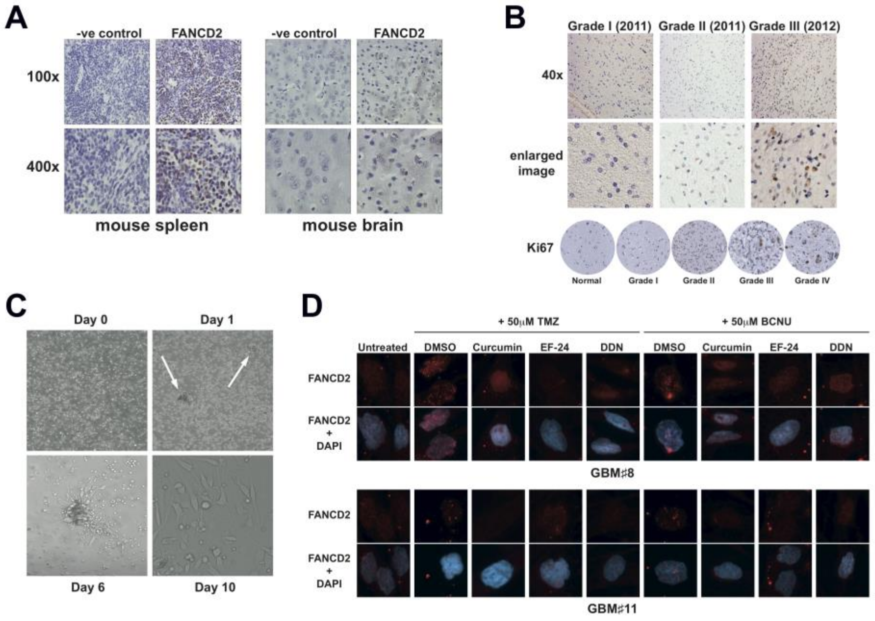

Supplementary Figure S1: A: Representative images of FANCD2 staining in FFPE mouse spleen (positive control) and brain (negative control) carried out as part of antibody optimisation studies. Shown are images at 100x and 400x magnification to allow visualisation across the section and in individual cells to show strong nuclear staining in the highly proliferative spleen cells. **B**: Upper panel; Representative FANCD2 expression in glioma specimens taken from an individual patient treated as Sheffield's Royal Hallamshire Hospital as described in the manuscript. Indicated are the tumour grade and year in which the sample was collected. Top panel shows a low power 40x magnification with the lower panel showing an enlarged section to facilitate visualisation of FANCD2 nuclear staining in the grade II and, in particular, the grade III tumour. Lower panel; examples of Ki67 staining in normal brain and tumours of indicated grade taken from the tissue microarray (Figure 1C). **C**: Representative phase contrast images showing the various stages of the development of primary glioma cultures derived from surplus clinical material collected at the neuro-oncology unit at Sheffield's Royal Hallamshire Hospital. White arrows indicate the formation of neuro-spheres as previously described [21, 22]. **D**: Representative images of immunofluorescence detection of FANCD2 nuclear foci in two independently derived primary glioma cultures as described in Figure 2D. Shown is FANCD2 staining alone or merged with DAPI staining to allow visualisation of FANCD2 foci within the nucleus. The relatively few red dots that reside outside the nucleus represent non-specific antibody staining and are common with this application.

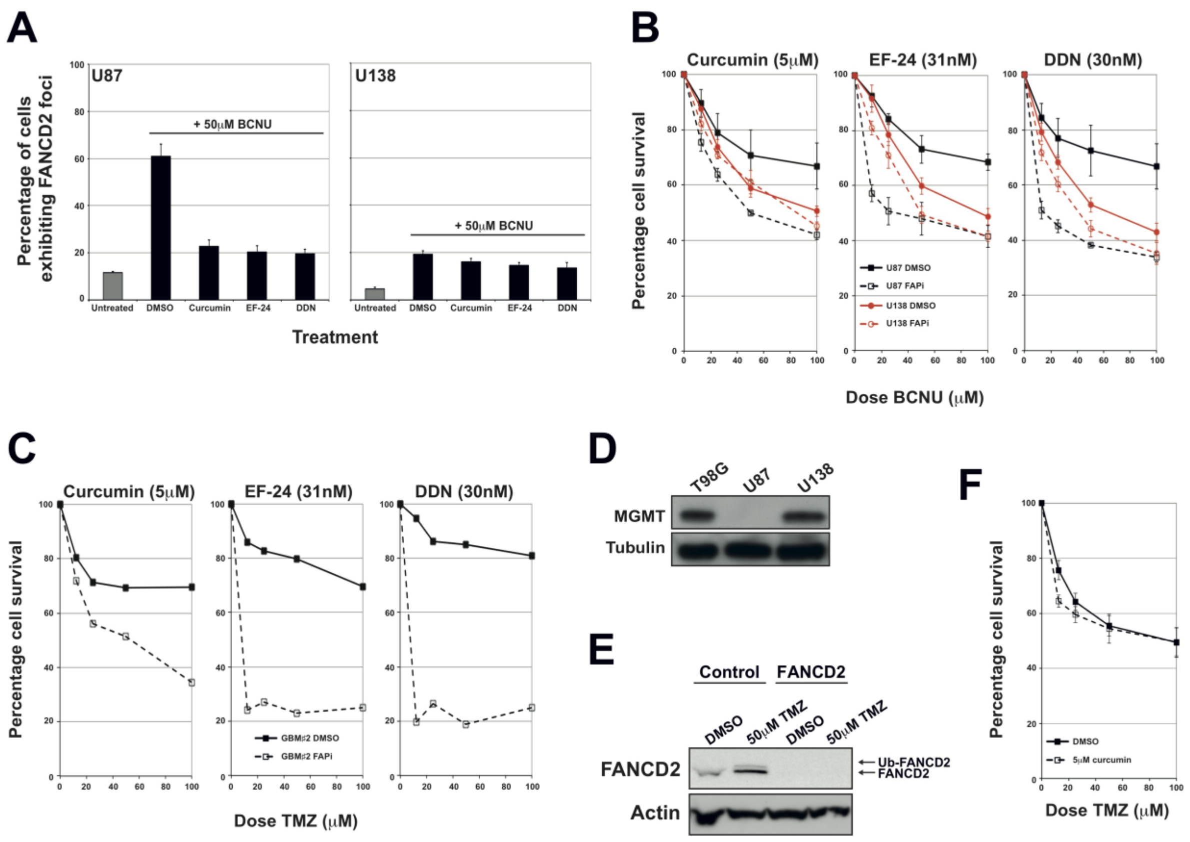

Supplementary Figure S2: A: Quantification of nuclear FANCD2 foci in U87 and U138 cells treated with the indicated FAPi and BCNU (DMSO serves as a negative control). Means were calculated from at least three independent experiments with error bars representing the standard deviation of the means. **B**: MTT BCNU cytotoxicity assays for U87 and U138 cells pre-treated with either DMSO or the indicated FAPi. Data shown was derived from at least three independent experiments and solid, dashed lines and error bars are as described in Figure 3A. **C**: MTT Temozolomide cytotoxicity assays for an additional primary glioma culture derived from surplus clinical material collected at the neuro-oncology unit at Sheffield's Royal Hallamshire Hospital. Solid, dashed lines and error bars are as described in Figure 3A. **D**: Western blot of T98G, U87 and U138 cells showing MGMT expression and tubulin (loading control). **E:** Western blot showing FANCD2 expression and activation (Ub-FANCD2) following Temozolomide treatment in an additional stable U87 cell line expressing FANCD2 directed shRNA (different target sequence to that used to generate the cell line shown in Figure 4). **F**: MTT Temozolomide cytotoxicity curves for the additional stable FANCD2-depleted (shRNA) U87 cell clone shown in panel E. Cells were pre-treated with either DMSO (solid line) or curcumin (dashed line) prior to TMZ treatment at the indicated dose. Data shown was derived from at least three independent experiments error bars representing the standard deviation of the means.
